# Synergistic suppression of ovarian cancer by combining NRF2 and GPX4 inhibitors: in vitro and in vivo evidence

**DOI:** 10.1186/s13048-024-01366-8

**Published:** 2024-02-23

**Authors:** Ning Li, Xingmei Jiang, Qingyu Zhang, Yongmei Huang, Jinbin Wei, Haitao Zhang, Hui Luo

**Affiliations:** 1 Laboratory of Obstetrics and Gynecology, Department of Obstetrics and Gynecology, Affiliated Hospital of Guangdong Medical University, Zhanjiang, 524001 Guangdong China; 2 Marine Biomedical Research Institute, the Key Lab of Zhanjiang for R&D Marine Microbial Resources in the Beibu Gulf Rim, Guangdong Medical University, Zhanjiang, Guangdong 524023 China; 3 The Marine Biomedical Research Institute of Guangdong Zhanjiang, Zhanjiang, Guangdong 524023 China; 4 Department of Hematology, Affiliated Hospital of Guangdong Medical University, Zhanjiang, 524001 Guangdong China; 5 Pharmaceutical College, Guangxi Medical University, Nanning, 530021 Guangxi China; 6 Department of Biochemistry and Molecular Biology, Guangdong Medical University, Zhanjiang, Guangdong 534023 China

**Keywords:** Ovarian cancer, NRF2, GPX4, Apoptosis, Ferroptosis

## Abstract

Ovarian cancer is a significant challenge in women's health due to the lack of effective screening and diagnostic methods, often leading to late detection and the highest mortality rate among all gynecologic tumors worldwide. Recent research has shown that ovarian cancer has an "iron addiction" phenotype which makes it vulnerable to ferroptosis inducers. We tested the combination of NRF2-targeted inhibitors with GPX4-targeted inhibitors in ovarian cancer through in vitro and in vivo experiment. The data showed that combination treatment effectively suppressed adherent cell growth, inhibited suspended cell spheroid formation, and restrained the ability of spheroid formation in 3D-culture. Mechanistically, the combination induced accumulation of ROS, 4-HNE, as well as activation of caspase-3 which indicates that this combination simultaneously increases cell ferroptosis and apoptosis. Notably, inhibition of GPX4 or NRF2 can suppress ovarian cancer spreading and growth in the peritoneal cavity of mice, while the combination of NRF2 inhibitor ML385 with GPX4 inhibitors showed a significant synergistic effect compared to individual drug treatment in a syngeneic mouse ovarian cancer model. Overall, these findings suggest that combining NRF2 inhibitors with GPX4 inhibitors results in a synergy suppression of ovarian cancer in vitro and in vivo, and maybe a promising therapeutic strategy for the treatment of ovarian cancer.

**Supplementary Information:**

The online version contains supplementary material available at 10.1186/s13048-024-01366-8.

## Introduction

Ovarian cancer (OC) is a highly lethal gynecologic malignancy with an overall 5-year survival rate only 47% [1]. Majority of cases diagnosed typically at advanced stages [2], and even though surgical intervention combined with chemotherapy can achieve high rates of complete remission of primary tumor, metastatic tumor is hardly removable and recurrence is inevitable [3]. Unlike other most solid tumor metastasis through blood or lymph system but tumor seeds spreading of inside peritoneal cavity, which make ovarian cancer special and difficult to diagnosis and treat. Chemotherapy is the major approach for unremovable metastatic ovarian cancer and hot perfusion is an efficacy strategy for elimination of ovarian cancer before or after surgical remove in clinical practice. However, over past decades there is no significant progress on developing of chemotherapeutics drugs for ovarian cancer. Therefore, new theory-based approaches are urgently needed to investigate and assessed on intervention ovarian cancer metastasis.

Ferroptosis is a unique form of programmed cell death, characterized by iron-dependent accumulation of lipid peroxides and byproduct of lipid peroxidation such as 4-hydroxynonenal (4-HNE) which are cytotoxic and cause cell membrane damage and cell death [4]. Recent evidence suggests that cancer cells exhibit heightened sensitivity to ferroptosis compared to normal cells, due to high lipid metabolic characteristics and genetic mutations [5–7]. In tumor cell, tumor suppressor gene RB1 mutation upregulates the levels of ACSL4 and enriches ACSL4-dependent arachidonic acid–containing (AA–containing) phospholipids which increase tumor cell vulnerability to ferroptosis [8]. The NF2/YAP/ACSL4 signaling pathway is activated in the low confluence of cells which suggests metastatic cancer cells could be vulnerable to ferroptosis inducer [9]. Cancer recurrence can be partially attributed to cancer stem cells (CSC) maintenance, a subpopulation characterized by self-renewal, high tumorigenicity, and drug resistance [10]. CSC of ovarian cancer cell have higher iron level demand which render them more susceptible to iron chelator or ferroptosis inducer. Recently, studies have demonstrated that ovarian cancer spheroids formation display a high dependence on iron [11, 12], indicating metastatic ovarian cancer cells are higher vulnerable to ferroptosis.

The lipid peroxidation removal system in cells is responsible for counteracting the harmful effects of lipid peroxidation. One key enzyme is glutathione peroxidase (GPX), which utilizes reduced glutathione (GSH) to convert lipid peroxides into their corresponding alcohols to neutralize their damaging effects [13]. GPX4 is the only member of the GPX family capable of converting lipid peroxides into non-toxic lipid alcohols to resist cell ferroptosis [14, 15]. Nuclear factor E2-related factor 2 (NRF2) is a transcription factor that serves as a master regulator of cellular redox homeostasis. In response to oxidative stress NRF2 bind to antioxidant response element (ARE) to regulate antioxidant gene such as Glucose-6-phosphate dehydrogenase (G6PD), Heme oxygenase-1(HO-1), GPX4 and cytochromes P450 (CYP) [16]. Under normal conditions, NRF2 is downregulated by Kelch-like ECH-associated protein 1 (KEAP1)-mediated ubiquitination and subsequent protein degradation [17]. However, during oxidative stress, KEAP1 undergoes structural alterations [18], resulting in decreased affinity for NRF2, enabling NRF2 nuclear translocation and upregulation of downstream antioxidant genes, including GPX4 [19]. Additionally, NRF2 influences GPX4 activity by modulating glutathione (GSH) uptake, a GPX4 substrate, by activation of the cystine/glutamate antiporter system (system Xc-) [16]. Emerging evidence implied that ovarian cancer cell encompasses the high oxidative pressure in peritoneal metastasis and activate the NRF2/GPX4 pathway to overcome lipid peroxidation [20]. Therefore, intervention NRF2/GPX4 pathway was believed can disrupt tumor cell redox homeostasis to prevent cancer progression.

Based on these evidences, we hypothesized that simultaneous inhibition of both GPX4 and NRF2 may be an effective way to suppress ovarian cancer metastasis. In this study, adherent, suspension and 3D culture cell were used to investigate the synergistic antitumor potential of inhibitors for both GPX4 and NRF2. Our results demonstrated that the combined treatment had a significantly greater effect than the individual drugs in reducing both monolayer cell viability and spheroid formation under suspension and 3D culture conditions. Moreover, the efficacy of this combination was further validated in an in vivo syngeneic mouse tumor model. Therefore, the combination therapy involving NRF2 inhibitors and GPX4 inhibitors holds great promise as a therapeutic approach for the treatment of ovarian cancer.

## Materials and Methods

### Reagents and Antibodies

The following reagents were used in this study: DMEM (Gibco, 8121334, USA), Fetal bovine serum (Biological Industries, 04–001-1ACS, Israel), penicillin–streptomycin liquid (Solarbio, P1400, China), trypsin (Solarbio, T1350, China), RIPA Lysis Buffer (Beyotime, P0013B, China), Bradford Protein Assay Kit (Beyotime, P0006C, China), anti-Tubulin (1:1000, CST, 2125, China), anti-actin (1:1000, Proteintech, 6009–1-Ig, China), anti-NRF2 (1:1000, CST, 12721 T, USA), anti-GPX4 (1:1000, CST, 52455S, USA), anti-p62 (1:1000, Proteintech, 00106301, China), anti-KEAP1 (1:1000, Proteintech, 10,503–2-AP, China), anti-HO-1 (1:1000, Proteintech, 10,701–1-AP, China), anti-4-HNE (1:100, MyBioSource, MBS808700, China), Cell Counting Kit-8 (Beyotime, C0038, China), PL-luciferase (Addgene, 21,471, USA), Annexin V-FITC/PI Apoptosis Kit (Biosharp, BL110A, China), GreenNuc™ Caspase-3 Assay Kit for Live Cells (Biosharp, C1168S, China), BODIPY™ 581/591 C11(Thermofisher, D3861, USA), Hoechst (Beyotime, C1011, China) and Luciferin (Beyotime, ST196, China). Trigonelline (Cat# B20521), Clobetasol propionate (Cat# B24212) and ML385 (Cat# S86700) were purchased from yuanye Bio-Technology Co, Ltd (Shanghai, China). RSL3 (Cat# A15865) and ML210 (Cat# A20475) were provided from AdooQ BioScience.

### Cell culture

The HM and OVCA429 human ovarian cancer cell lines were kindly gifted by prof. Tsz on LEE in University of Macau. ID8 cell was purchased from Fenghui biotech (Changsha, China). ID8-luc cell line was generated by infection cell with luciferase lentivirus particles and select with puromycin (1 μg/mL) for 3 days. The cell lines were maintained in DMEM supplemented with 10% fetal bovine serum (Biological Industries, USA) and 1% penicillin–streptomycin in a humidified incubator at 37 °C with 5% CO_2_. All cell lines' origins have been validated through STR testing, and routine assessments of cell growth conditions are conducted to monitor for mycoplasma contamination.

### Cell viability assay

HM and OVCA429 cells were seeded into 96-well plates in density of 2.0 × 10^3^. After 24 h of incubation, the cells were exposed to drugs and cell viability was tested by CCK8 assay. Briefly, 10 μL CCK8 was added to each well and incubated for 2 h and readout the optical density (OD) at 450 nm using a microplate reader. Cell viability was calculated using the formula: (%) = [D-D_0_]/[‾D-D_0_] × 100%. D was the OD value of the experimental group, D_0_ represented the OD of the blank control and‾D was the mean OD value of the control group.

### PI staining

2000 cells per well were pre-seeded in a 96-well plate. After indicated drug treatment, the medium was replaced by a fresh medium containing final concentration of 50 μg/mL propidium iodide (PI, Beyotime, ST511, China) and 10 μg/mL Hoechst (Beyotime, C1011, China). After 30 min incubation, the cells were observed underfluorescence microscope and photographed image within 1 h.

### Spheroids formation assay

The method can refer to previous studies [11, 21]. Briefly, 6-well plate was rinsed with 0.5% agarose and 3000 cells were seeded in each well and treated with drugs. After 7–10 days of growth, the formed spheroids were gently transferred to a new plate to grow for another 7–10 days. The colonies were washed twice with PBS and fixed with 4% paraformaldehyde for 10 min and stained with 0.1% crystal violet solution for 15 min. The colonies were then photographed and counted using Image J software.

### 3D culture

Cells were collected and resuspended in 5% Matrigel (Corning, 356234, USA) in phenol red-free medium (PM150223, Procell, China). 96-well plated pre-rinsed with 0.5% agarose gel and 300 cells in 50 μL were seeded in 96-well plate per well. 1% Matrigel was used to prepare the 2 × concentration of drugs and additional 50 μL 2 × drug medium was added to above 3D culture cell. The medium was replaced every three days.

### Calcein AM/PI staining

After the formation of 3D spheroids (consisting of at least 50 cells), the Calcein AM/PI staining solution (C2015, Beyotime, China) was employed. 100 μL of the staining solution was added to each well, followed by incubation at room temperature in the dark for 30 min. After the incubation, the cells were examined and imaged using fluorescence microscopy within 1 h.

### Apoptosis assay

Cells were seeded into 12-well plates and treated with varying drug concentrations in fresh medium for 48 h. After treatment, cells were harvested and suspended in staining buffer. According to the apoptosis assay kit (BL110A, Biosharp, China) instruction protocol, 100,000 cells per sample were employed in this assay and PI (10 μL) and Annexin V-FITC (5 μL) were added to each sample and incubated in the dark at 37℃ for 10 min. Flow cytometry (Cytek, China) was used to analyze the samples.

### ROS analysis

Cells were seeded into 12-well plates and treated with varying drug concentrations in fresh medium for 48 h. After treatment, cells were stained with 1.5 μM BODIPY C11 (Thermofisher, D3861, USA) for 30 min in the dark at 37℃ incubators. Cells were harvested into corresponding tubes and washed with PBS twice. Cells were resuspended in 500 μL PBS and analyzed using a flow cytometer (Cytek, China) and FlowJo software was employed to analyses.

### Caspase-3 activity assay

Caspase-3 expression was measured using the GreenNuc™ Caspase-3 Assay Kit (Biosharp, C1168S, China) and flow cytometry. Cells were seeded into a 12-well plate and treated with medium containing different drug concentrations for 48 h. After treatment, cells were collected, washed twice, and incubated with 5 μM dyes for 30 min at room temperature protected from light. Flow cytometry was performed to analyze Caspase 3 activity.

### Western blotting analysis

Cells/spheroids were collected and washed twice with PBS, then lysed using RIPA buffer and quantified with a Detergent-Compatible Bradford Protein Assay Kit. Protein samples (20–30μg) were separated by gel electrophoresis and transferred to PVDF membranes. After blocking with 5% non-fat milk, membranes were incubated with primary antibodies (dilution 1:1000) overnight at 4℃. HRP-conjugated secondary antibodies (dilution 1:5000) were used and the aim protein were visualized by adding ECL substrate and the protein signal were captured in an illuminance imaging system (Sally Sue, Proteinsimple, USA). The resulting images were analyzed using ImageJ.

### qPCR analysis

Cell extracts were prepared using the AG RNAex Pro kit (Accurate Biology, AG21101, China) and RNA was reverse-transcribed into cDNA using the HiScript III RT SuperMix for qPCR (+ gDNA wiper) kit (Vazyme, R323-01, China). Real-time qPCR was performed on a Analytik Jena real-time PCR system (Jena, Germany) using ChamQ Universal SYBR qPCR Master Mix (Vazyme, Q711, China). The qPCR primer sequences used are listed below and expression was normalized to β-actin.

**Table:** 

GENE		Primer Sequence
*ACTB*	Forward	AGATGTGGATCAGCAAGC
	Reverse	TCATCTTGTTTTCTG CGC
*NFE2L2*	Forward	CACATCCAGTCAGAAACCAGTGG
	Reverse	GGAATGTCTGCGCCAAAAGCTG

### Immunofluorescence staining

After treatment, cells were fixed with 4% paraformaldehyde, blocked with 0.1% Triton-X100 and 1% BSA in phosphate-buffered saline (PBS), and incubated with primary antibody (1:100) overnight at 4℃. Cells were then incubated with secondary fluorescent antibodies (1:200) and mounted with Antifade Mounting Medium (P0133, Beyotime, China). Images were captured using an Olympus confocal laser scanning microscope system. The fluorescence intensity of the indicated proteins was quantified using ImageJ software.

### shRNA mediated gene silencing

To generate the gene stable knockdown cell, target cells were infected by lentivirus generated in HEK-293T cells and selected out the gene knockdown cell using puromycin (1 μg/mL) for 48 h. shNRF2 interference sequences used in this study are: shNRF2-1: 5′-GCCCATTGATGTTTCTG ATCT-3; shNRF2-3: 5′- GCA GTTCAATGAAGCTC AA CT-3’.

### The mouse ovarian cancer peritoneal spreading model

The animal experiment followed the Declaration of Helsinki guidelines and was approved by the Guangdong Medical University Animal Ethical Committee (GDY2102330). 5-week-old female C57BL/6 mice were obtained from Yancheng Biological Company and housed in an SPF laboratory at 26℃ with free access to water and food. Mice were intraperitoneally injected with 5 × 10^6^ ID8-Luc-puro cells in 200 μL PBS. On the seventh day after cell inoculation, mice were treated with 1 mpk trigonelline, 30 mpk ML385, 0.5 mpk clobetasol propionate, 3 mpk RSL3, 5 mpk ML210 and their respective combinations, while 10% DMSO + 40% PEG300 + 5% Tween-80 + 45% saline was given as a control. Body weight was measured every 5 days. After treatment, mice were injected with pentobarbitone (1 mg/mice) and D-luciferin (3 mg/mice) successively, and luminescence signals were observed within 15 min using the *In-vivo* Xtreme live imaging system (Bruker, USA). Illuminance intensity was normalized and analyzed using Bruker MI SE software.

### Statistical analysis

The results are presented as mean ± SEM from at least three independent experiments, and statistical analysis was performed using GraphPad 8.0. Two-tailed, unpaired Student’s t-test or ANOVA with Tukey post-hoc analysis was used to determine statistical significance. For animal experiments, at least three to six animals per group were used in each of two independent experiments. Statistical significance was determined using Student’s t-test and *p* < 0.05 was considered significant. Significance levels were represented as *p < 0.05, **p < 0.01, ***p < 0.001 and ****p < 0.0001.

## Results

### NRF2 upregulated in ovarian cancer spheroid formation and promotes cancer metastasis

**Fig.1:**
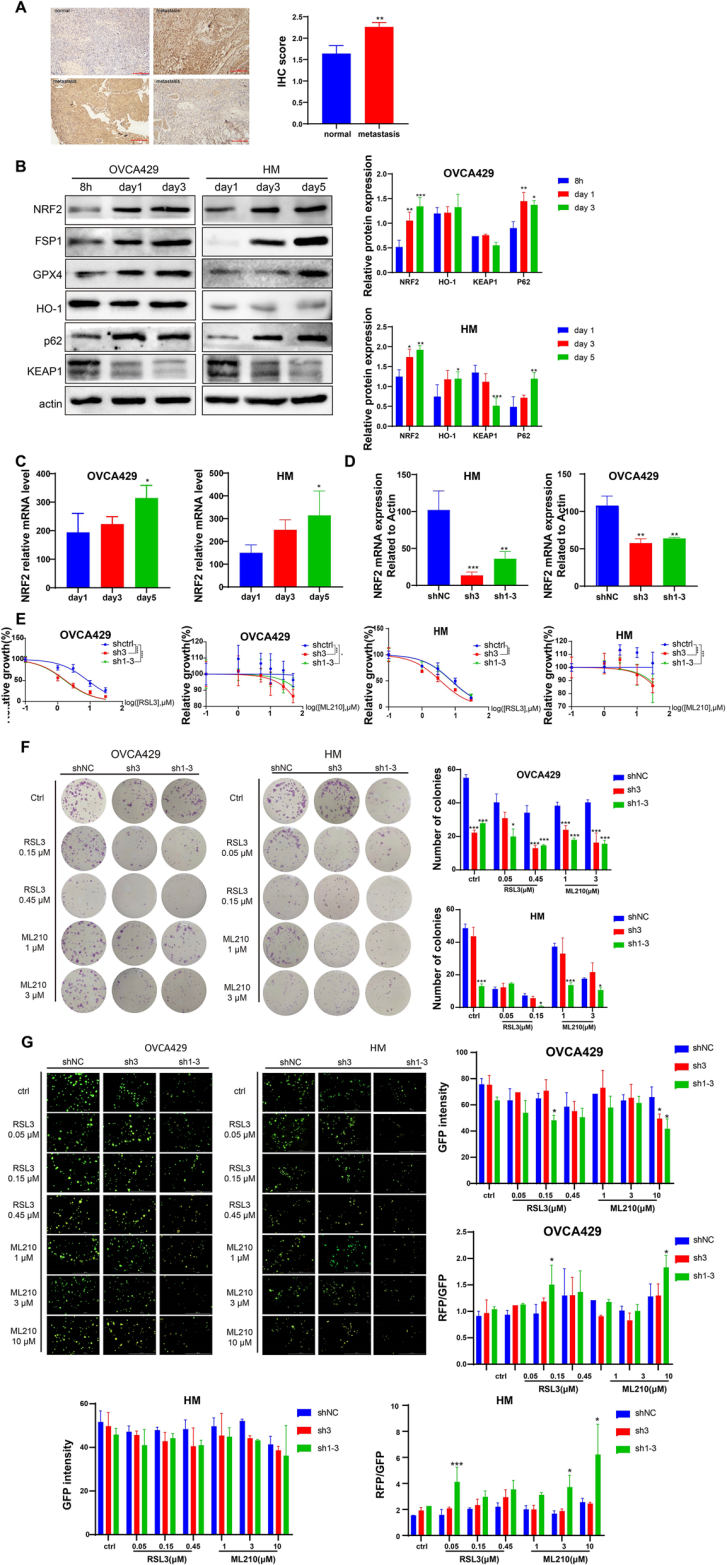
NRF2 KD increases the sensitivity of adherent, suspending and 3D cells to GPX4 inhibitors. **A** IHC scores of NRF2 in primary tumor and metastasis tumors from 51 patients. Primary tumor IHC scores mean ± SD = 1.82 ± 0.76; Metastasis tumor IHC scores mean ± SD = 2.22 ± 0.63). **B** Cell culture in suspension for 8 h, 1 day, 3 days and 5 days the protein level of NRF2, KEAP1,p62, HO-1 and GPX4 in HM and OVCA429 cells was measured by immune blot. **C** Quantitative real-time PCR was performed to assess mRNA level of *NFE2L2* in HM and OVCA429 cells after suspension growth for 1 day, 3 days and 5 days. **D** Gene knockdown confirmation after transfected with shRNA targeting NRF2 the *NFE2L2* mRNA measured by Quantitative real-time PCR. **E** HM and OVCA429 cells with or without *NFE2L2* knockdown were treated by GPX4 inhibitors for 48 h the cell viability was measured by CCK8 assay. **F** The inhibitory effect of GPX4 inhibitors on spheroid formation of HM and OVCA429 cells transfected with shNC and shNRF2 was evaluated. **G** Calcein AM/PI staining was used to assess the cell deaths on 3D spheroid formation of HM and OVCA429 after treatment of GPX4 inhibitor and knockdown of NRF2. Each experiment was performed in triplicate. Statistical significance was represented as *p < 0.05, ** *p* < 0.01 and *** *p* < 0.001 compared to the control group

To investigate the NRF2 gene expression pattern in clinical sample, transcriptomics data were extracted from TCGA database and we found that *NFE2L2* expression was significantly upregulated in ovarian cancer (Fig. 1A). To evaluate NRF2 expression in primary and metastatic tumors, we examined NRF2 expression in 51 patients by immunohistochemistry and found that NRF2 levels were significantly higher in the metastasis tumor group than in the normal ovary group (Fig. 1A). Hence, we hypothesized that upregulation of NRF2 in ovarian cancer promotes cancer cell metastasis in response to the overwhelming ROS harsh environment in the peritoneal cavity. We first examined the expression of NRF2 in the process of spheroid formation and found that NRF2 protein levels and *NFE2L2* mRNA expression levels were upregulated (Fig. 1B-C). We also found KEAP1 was downregulated while p62 was upregulated as spheroids formation. Furthermore, the NRF2 downstream transcriptional target genes HO-1 and GPX4 were both upregulated (Fig. 1B), suggesting that NRF2 is involved in ovarian cancer metastasis.

To test the hypothesis that inhibition of NRF2 could improve the anti-tumor effect of ferroptosis inducers in ovarian cancer cells, we knocked down NRF2 through stable transduction of shRNA targeting *NFE2L2* (Fig. 1D and s-Fig. 1A). Our data indicated that knockdown of NRF2 increased cell sensitivity to GPX4 inhibitors RSL3 and ML210 (Fig. 1E). Additionally, we found that knockdown of NRF2 resulted in a decrease in the ability of spheroids formation in suspension and 3D culture scenarios (Fig. 1F-G and s-Fig. 1B). Our findings demonstrated that NRF2 promotes ovarian cancer survival and growth and targeting NRF2 with GPX4 inhibitors could exert a synergistic effect on ovarian cancer cells.

## Synergistic effect of NRF2 inhibitors and GPX4 inhibitors on adherent, suspending and 3D ovarian cancer cell growth

**Fig. 2:**
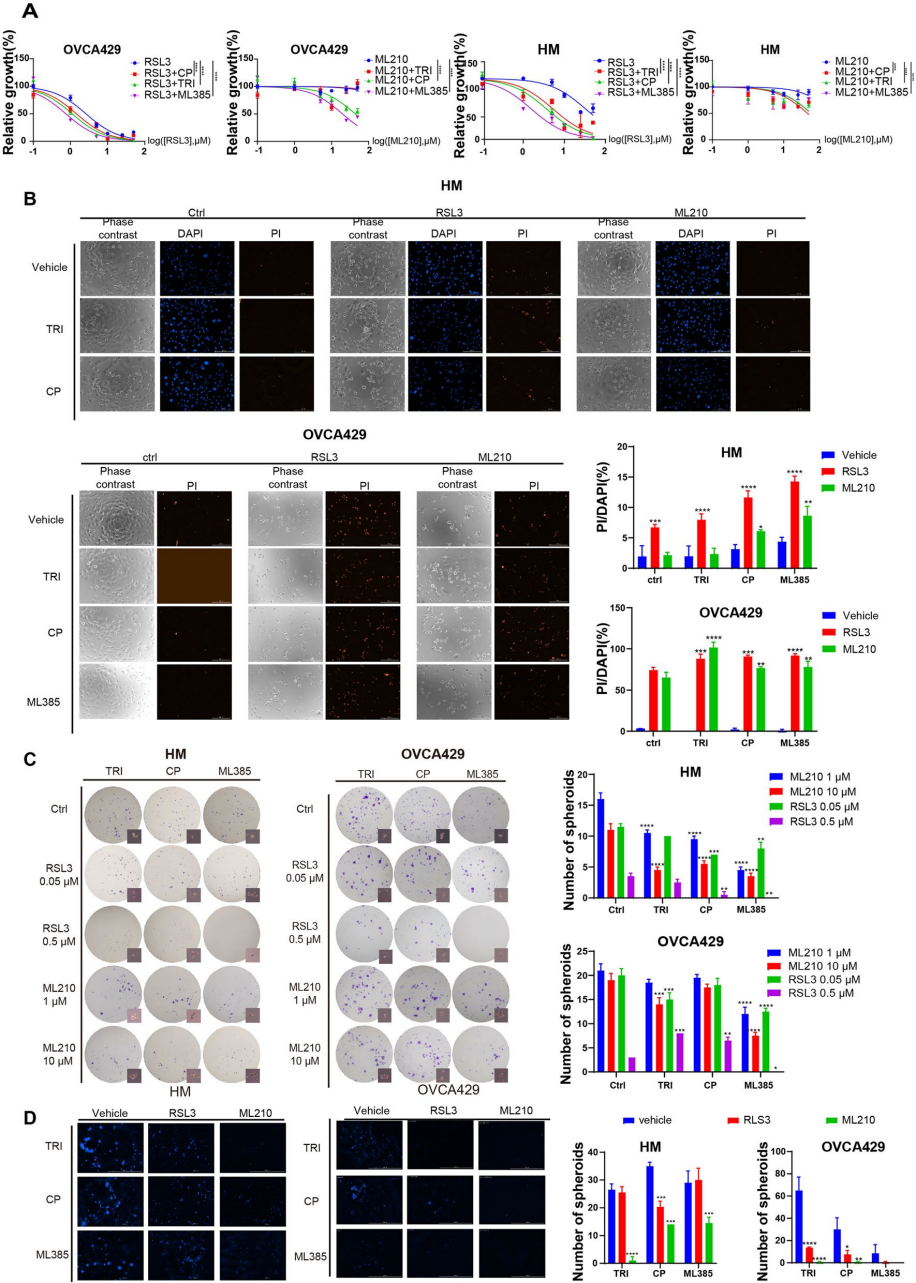
The antitumor ability of mono-treatment and combination therapy in vitro. **A** Cytotoxicity of drugs on HM and OVCA429 cells was measured by CCK8. **B** PI (50 μg/mL) and Hoechst (10 μg/mL) staining was used to detect the dead cell after treatment. **C** The suppression effect of combination treatment on spheroid formation was evaluated in HM and OVCA429 cells. **D** The inhibitory effect of combination treatment on 3D spheroid formation was evaluated by Hoechst staining (10 μg/mL). Each data point represents the mean ± SEM of three independent experiments. ** *p* < 0.01, *** *p* < 0.001 and **** *p* < 0.0001 indicate statistical significance when compared to the control group

To investigate the anti-cancer effect of NRF2 inhibitors combined with GPX4 inhibitors, we chose three NRF2 inhibitors (trigonelline (TRI), Clobetasol propionate (CP), and ML385) and two GPX4 inhibitors (RSL3 and ML210). Our results showed that RSL3 and ML210 reduce cell viability in a dose-dependent manner in HM and OVCA429 ovarian cancer cell lines (s-Fig. 2A). However, all three NRF2 inhibitors did not show significant cytotoxicity on the tested cells (s-Fig. 2B). Additionally, the dead cells after treatment were detected by PI staining, and the results verified that NRF2 inhibitors did not increase the PI-positive cell rates (s-Fig. 2C), except for ML385 on HM cells. Despite the toxicity of ML385 on HM cells, it induced less than 6% cell death even at 50 µM (s-Fig. 2C). Combination use of drugs usually exert a synergistic effect. In our study, the combination NRF2 inhibitors can further suppress cell viability and induce cell death when compared to the single use of GPX4 inhibitors in HM and OVCA429 cancer cell lines (Fig. 2A-B). Functional assay results indicated that combination treatment has a significant synergistic effect in inhibiting spheroid formation ability (Fig. 2C). Furthermore, the combination of these drugs reduced spheroid growth in 3D culture, validating this synergistic effect (Fig. 2D). We also conducted a sensitivity analysis of organoids to NRF2 inhibitor CP and GPX4 inhibitor RSL3 and found that metastasis cancer cells were more sensitive to ferroptosis inducers compared to primary tumor cancer cells (s-Fig. 2D), which consistent with our hypothesis.

## Combination of NRF2 inhibitors and GPX4 inhibitors synergistically increases cell ferroptosis

**Fig. 3:**
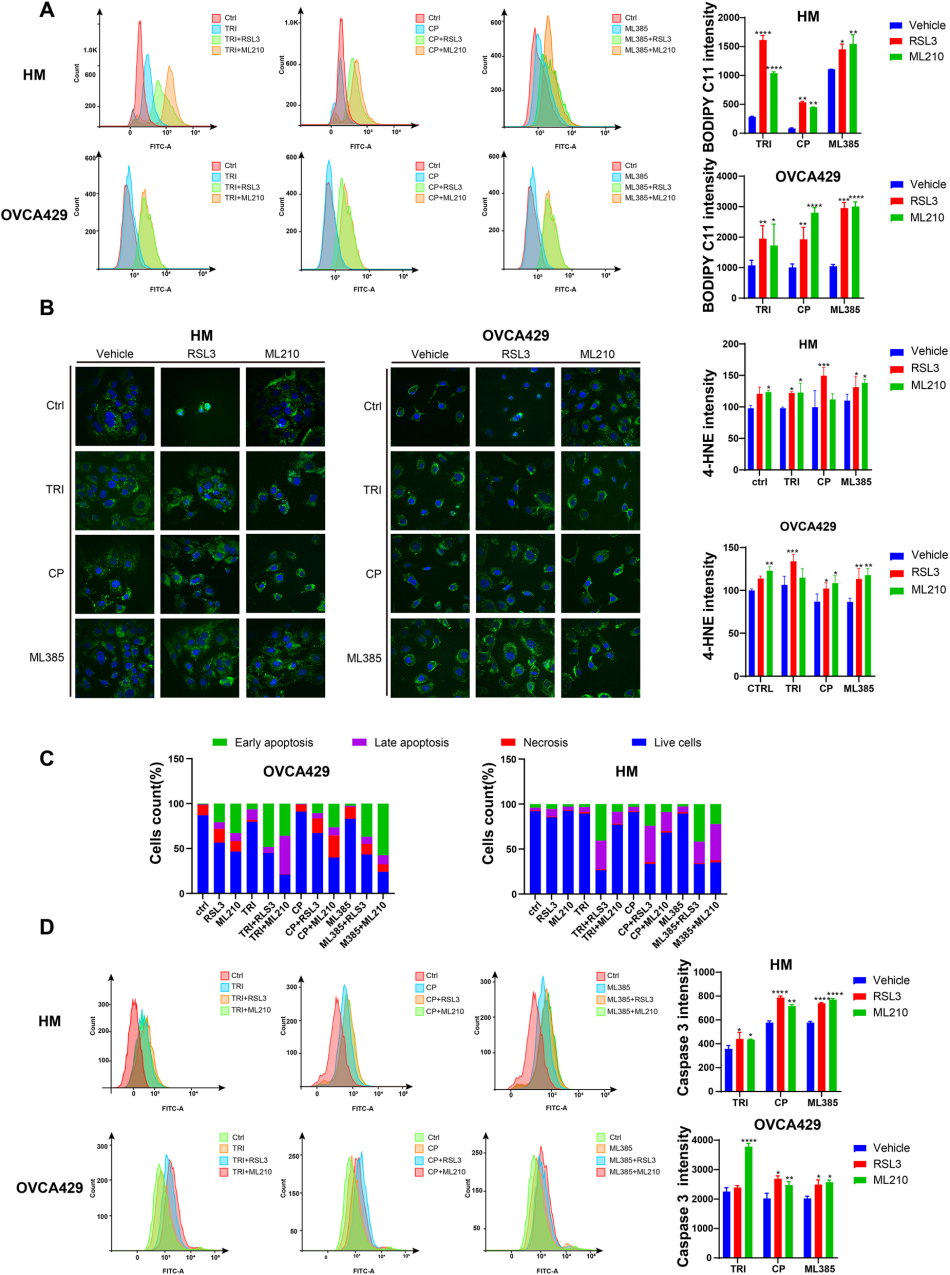
Combination treatment aggravates ROS and 4-HNE accumulation and induces apoptosis. **A** lipid peroxides in HM and OCVA429 cells after individual or combination treatment was measured by BODIPY C11 staining and flow cytometry analysis. **B** confocal imaging (left) and quantification (right) of 4-HNE in HM and OVCA429 cells after individual and combination treatments. **C** Annexin V-FITC/PI staining and flow cytometry analysis was used to quantify cell apoptosis induced by drug treatment in HM and OVCA429 cells. The results were presented as stacked bars. **D** Flow cytometry was performed to assess the activity of caspase-3 in HM and OVCA429 cells after drug treatment for 48 h. Each experiment was performed in triplicate. Statistical significance was represented as *p < 0.05, ** *p* < 0.01 and *** *p* < 0.001 compared to the control group

To investigate the mechanism of combination NRF2 inhibitor and GPX4 induced cell death, 2e hypothesized that targeting NRF2 and GPX4 would disrupt the homeostasis of ROS resulting in cell ferroptosis. To examine the effect of the combination treatment on lipid ROS levels, we measured ROS levels in the treated cells using flow cytometry. As expected, we observed an increased ROS level in the combined treatment group (Fig. 3A). 4-Hydroxynonenoic acid (4-HNE) is an end product of lipid peroxidation and a marker for lipid peroxidation. We also detected the abundance of 4-HNE using an Immunofluorescence assay. The combination treatment increased the 4-HNE content in ovarian cancer cells (Fig. 3B). It’s worth noting that the characteristic of ferroptosis is cell swelling and content leakages, but we also observed a cell shrinkage phenotype, indicating that cell apoptosis may be involved in the combination of these two targeted inhibitors. We then detected cell apoptosis using the Annexin V-FITC/PI apoptosis staining kit. The results showed that the combined usage significantly increased apoptosis in HM and OVCA429 cells (Fig. 3C). In addition, we measure the activity of caspase-3, an executor of cell apoptosis. The results confirmed combination treatment increase caspase-3 activity when compared to individual drug treatment (Fig. 3D).

## Synergistic antitumor efficacy in mice

**Fig. 4:**
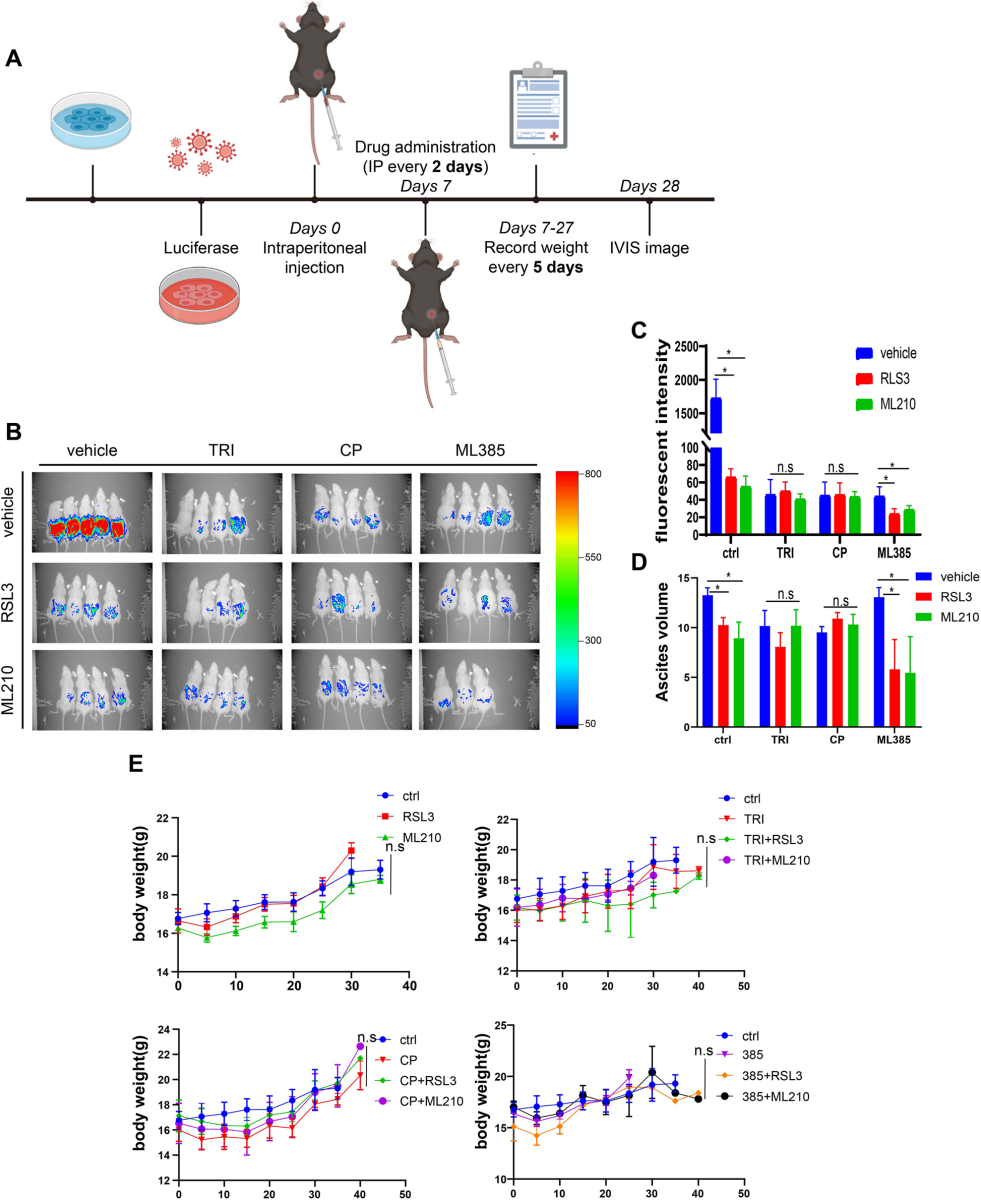
Combination treatment improves the antitumor activity in vivo. **A** Flow chart of animal experiments. **B** In vivo imaging was used to monitor the spread of ID8-Luc cells in mice. **C** Bruker MI SE software was used to quantify fluorescence intensity emitted from ID8-luc cells. **D** The volume of ascites produced in each group was measured after the mice were sacrificed. Each group consisted of at least three mice in every experiment and two independent experiments were performed. **E** The body weight of mice was monitored every five days since the first administration. Statistical significance was represented as *p < 0.05, **p < 0.01 and ***p < 0.001 compared with the control group

To evaluate the anti-cancer effects of combination treatment in vivo, a syngeneic ovarian cancer mouse model was used. ID8-Luc cells were intraperitoneally injected into C57BL/6 mice, allowing the ovarian cancer cells to grow for 7 days before grouping. 49 mice were randomly divided into 12 groups (5 mice in vehicle group, and 4 mice in every inhibitor treatment group). The drugs were intraperitoneally administrated every other day for a total of 10 administrations. After the final treatment, in vivo imaging technology was performed to assess the anti-cancer effect of the treatments (Fig. 4A-B). Our results showed that individual treatment had a significant metastasis inhibition effect compared to the control group, without affecting the body weight of the mice, except for the GPX4 inhibitor ML210, which slightly reduced the body weight of mice (*p* = 0.1026, Fig. 4E). However, when GPX4 inhibitor ML210 and RSL3 were combined with NRF2 inhibitors TRI and CP, they did not exhibit a synergistic effect on tumor growth and ascites production reduction. In contrast, a combination of ML385 with RSL3 or ML210 significantly suppressed ovarian cancer growth and ascites production without affecting the body weight of mice (Fig. 4B-E). Our study suggests that combining NRF2 inhibitor ML385 with RSL3 or ML210 may be an effective treatment strategy for ovarian cancer.

## Discussion

Ovarian cancer (OC) is one of the most lethal gynecological malignancies worldwide [22]. Shockingly, the number of deaths caused by ovarian cancer is projected to reach 250,000 by 2035 [23]. Therefore, it is imperative to develop new and effective methods for the treatment of ovarian cancer to combat this highly fatal disease.

Apoptosis, a form of programmed cell death, can be triggered via internal or external pathways [24]. The internal pathway involves signal molecules induced by intracellular events such as oxidative stress [25], while the external pathway involves ligands binding to death receptors on the cell membrane [26]. Ferroptosis, a distinct form of regulated cell death, is caused by a depletion of glutathione [27], leading to increased ROS levels [28]. Carcinogenesis often leads to the development of resistance to cell apoptosis in transformed cells [29]. Therefore, triggering both apoptosis and ferroptosis simultaneously would more effectively promote tumor regression [30]. NRF2 is the master transcription factor responsible for regulating cellular resistance to oxidants. Combining GPX4 and NRF2 inhibitors further impairs antioxidant processes, leading to an overload of reactive oxygen species (ROS) and resulting in overwhelming oxidative stress and cell death [31, 32]. Several previous studies have reported that silencing NRF2 sensitizes tumor cells to GPX4 inhibitors in head and neck cancer as well as acute myeloid leukemia [33, 34], indicating that NRF2 renders tumor cells resistant to ferroptosis. One study reported that the NRF2 inhibitor trigonelline increases the sensitivity of head and neck cancer cells to GPX4 inhibitors such as RSL3 or ML162 [34]. The combination of the NRF2 inhibitor ML385 with RSL3 synergistically targets acute myeloid leukemia [33]. Besides, ROS production in mitochondria leads to disturbances in the electron transport chain and ultimately triggers apoptosis via the activation of caspase-3 [35, 36] and our data showed the combination of NRF2 and GPX4 inhibitors also activates caspase-3. Our work indicated a clinical significance by targeting NRF2 and GPX4 which simultaneously trigger cell ferroptosis and apoptosis and synergistically eliminate ovarian cancer cells.

Our in vivo experimental data presents compelling evidence supporting the inhibitory effects of NRF2 or GPX4 inhibition on the growth and dissemination of ovarian cancer within the peritoneal cavity of mice. This observation underscores the potential significance of disrupting ROS homeostasis through targeted inhibition of NRF2 or GPX4, proposing a promising strategy for impeding ovarian cancer metastasis. The individual efficacy of NRF2 and GPX4 inhibitors is noteworthy, as their singular application demonstrates substantial inhibitory effects. Specifically, inhibiting NRF2 with ML385 exhibits a remarkable synergy effect at higher dosages when compared to GPX4 inhibition alone. This intriguing outcome suggests a potential interconnection between NRF2 and GPX4, hinting at a shared pathway in mitigating ovarian cancer progression. Moreover, it also suggests that it’s imperative to rigorously evaluate the pharmacokinetics of these inhibitors and enhance the bioavailability of these inhibitors to guarantee their successful advancement in clinical trials. However, it also needs to recognize the complexity of cellular responses and potential compensatory mechanisms. The lack of testing on compensation pathways, such as FSP1/CQ10 and DHODH/CoQH2, in this study highlights a valuable avenue for further investigation. Exploring these compensation pathways could provide deeper insights into the interconnected regulatory networks that govern ROS elimination, potentially uncovering additional targets for therapeutic intervention [32, 37], which had not been tested in this study and could be a potential strategy for further examination.

In summary, our findings suggested that combining NRF2 inhibitors and GPX4 inhibitors leads to synergistic inhibition of the growth of ovarian cancer by induction of ferroptosis as well as apoptosis. Our study strengths include its clinical relevance, mechanistic insights into apoptosis and ferroptosis, and in vivo evidence of inhibitory effects. Synergistic effects and anti-metastasis activity are highlighted, offering potential therapeutic benefits. However, limitations include the unexplored compensation pathways and the need to test pharmacokinetics of inhibitors and enhance their bioavailability. The study's applicability in diverse microenvironments and tumor heterogeneity is not fully explored, and the transition from preclinical to clinical settings may face challenges. Despite these limitations, the findings provide promising evidence for a novel therapeutic strategy against ovarian cancer, emphasizing the importance of further investigation and careful consideration of translational complexities.

### Supplementary Information

**Additional file 1: Supplementary Figure 1.** (A) NRF2 protein expression level in the indicated cell lines after knockdown by shRNA transfection were measured by western blotting. (B) The inhibitory effect of GPX4 inhibitors on 3D spheroid formation of HM and OVCA429 cells, which had been knockdown of NRF2, was assessed using Hoechst staining at a concentration of 10 μg/mL. Each experiment was performed in triplicate. Statistical significance was represented as *p<0.05, ** *p* < 0.01, *** *p* < 0.001 compared to the control group. **Supplementary Figure 2.** (A) Cell viability of HM and OVCA429 cells was determined using a CCK-8 assay after treatment with GPX4 inhibitors RLS3 and ML210 for 48 h. (B) Cell viability of HM and OVCA429 cells was determined using a CCK-8 assay after treatment with NRF2 inhibitors TRI, CP and ML385 for 72 h. (C) PI (50 μg/mL) and Hoechst (10 μg/mL) staining was used to assess the cytotoxic of the NRF2 inhibitors TRI, CP and ML385 treatments on HM and OVCA429 cells. (D) Cell viability of organoids was determined using a CCK-8 assay after treatment with indicated agents for 24 h (Left panel: organoids formed by metastatic tumor; right panel: organoids formed by primary tumor). Each experiment was performed in triplicate. Statistical significance was represented as *p<0.05, ** *p* < 0.01, *** *p* < 0.001 compared to the control group.

## Data Availability

The authors confirm that the data supporting the findings of this study are available within the article. Declarations Competing interests All authors agree there is no conflict interest exist.
