# Supplementary material for: Synergistic suppression of ovarian cancer by combining NRF2 and GPX4 inhibitors: in vitro and in vivo evidence
Source: J Ovarian Res. 2024 Feb 23;17:49. doi: 10.1186/s13048-024-01366-8 (PMC10885431; doi:10.1186/s13048-024-01366-8)
Supplement: Supplementary file 1 — Additional file 1: Supplementary Figure 1. (A) NRF2 protein expression level in the indicated cell lines after knockdown by shRNA transfection were measured by western blotting. (B) The inhibitory effect of GPX4 inhibitors on 3D spheroid formation of HM and OVCA429 cells, which had been knockdown of NRF2, was assessed using Hoechst staining at a concentration of 10 μg/mL. Each experiment was performed in triplicate. Statistical significance was represented as *p<0.05, ** p < 0.01, *** p < 0.001 compared to the control group. Supplementary Figure 2. (A) Cell viability of HM and OVCA429 cells was determined using a CCK-8 assay after treatment with GPX4 inhibitors RLS3 and ML210 for 48 h. (B) Cell viability of HM and OVCA429 cells was determined using a CCK-8 assay after treatment with NRF2 inhibitors TRI, CP and ML385 for 72 h. (C) PI (50 μg/mL) and Hoechst (10 μg/mL) staining was used to assess the cytotoxic of the NRF2 inhibitors TRI, CP and ML385 treatments on HM and OVCA429 cells. (D) Cell viability of organoids was determined using a CCK-8 assay after treatment with indicated agents for 24 h (Left panel: organoids formed by metastatic tumor; right panel: organoids formed by primary tumor). Each experiment was performed in triplicate. Statistical significance was represented as *p<0.05, ** p < 0.01, *** p < 0.001 compared to the control group. [file 13048_2024_1366_MOESM1_ESM.pptx]

## Slide 1
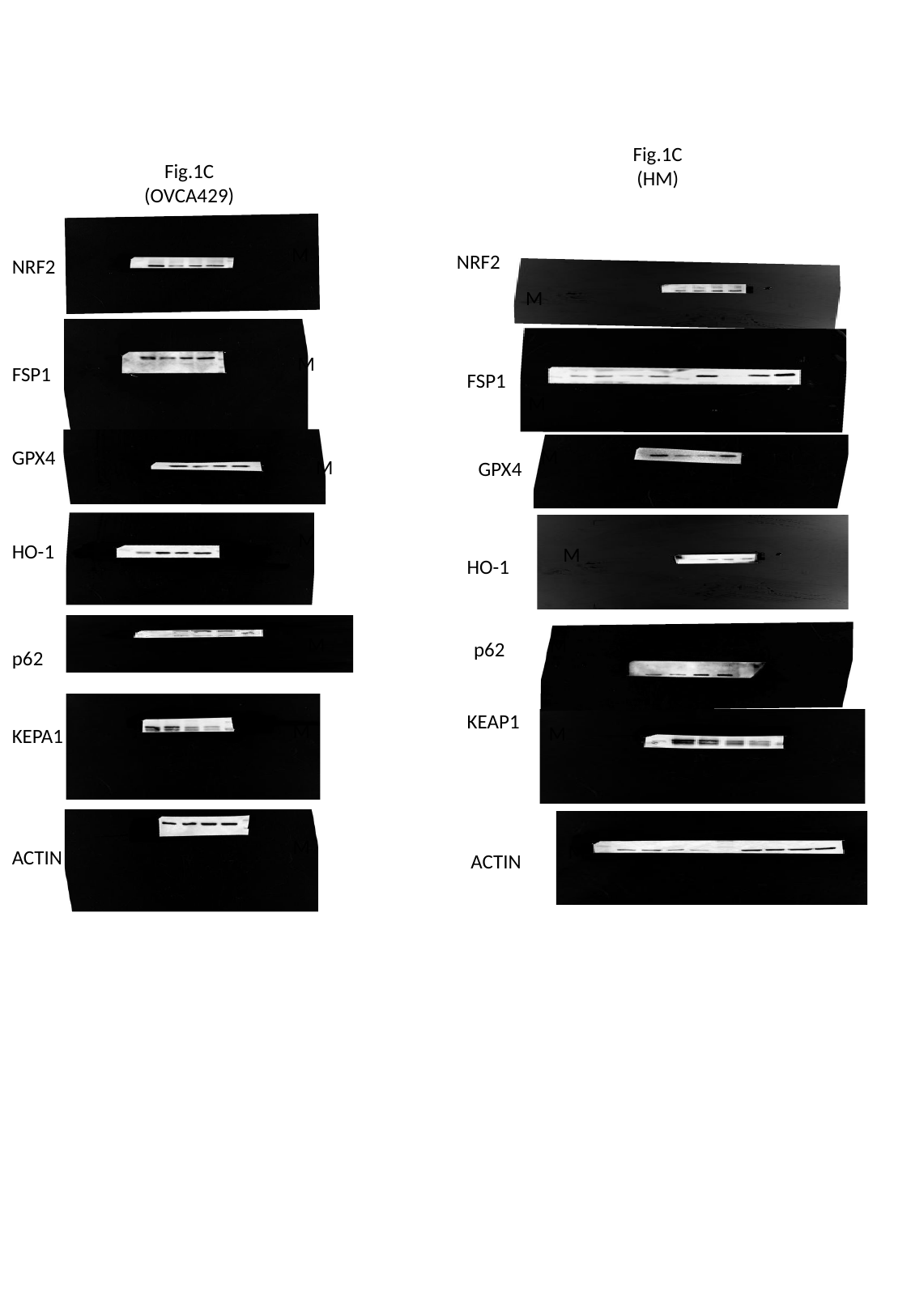

Fig.1C
(HM)
Fig.1C
(OVCA429)
M
NRF2
NRF2
M
M
FSP1
FSP1
M
M
GPX4
M
GPX4
M
HO-1
M
HO-1
M
M
p62
p62
KEAP1
M
M
KEPA1
M
M
ACTIN
ACTIN

## Slide 2
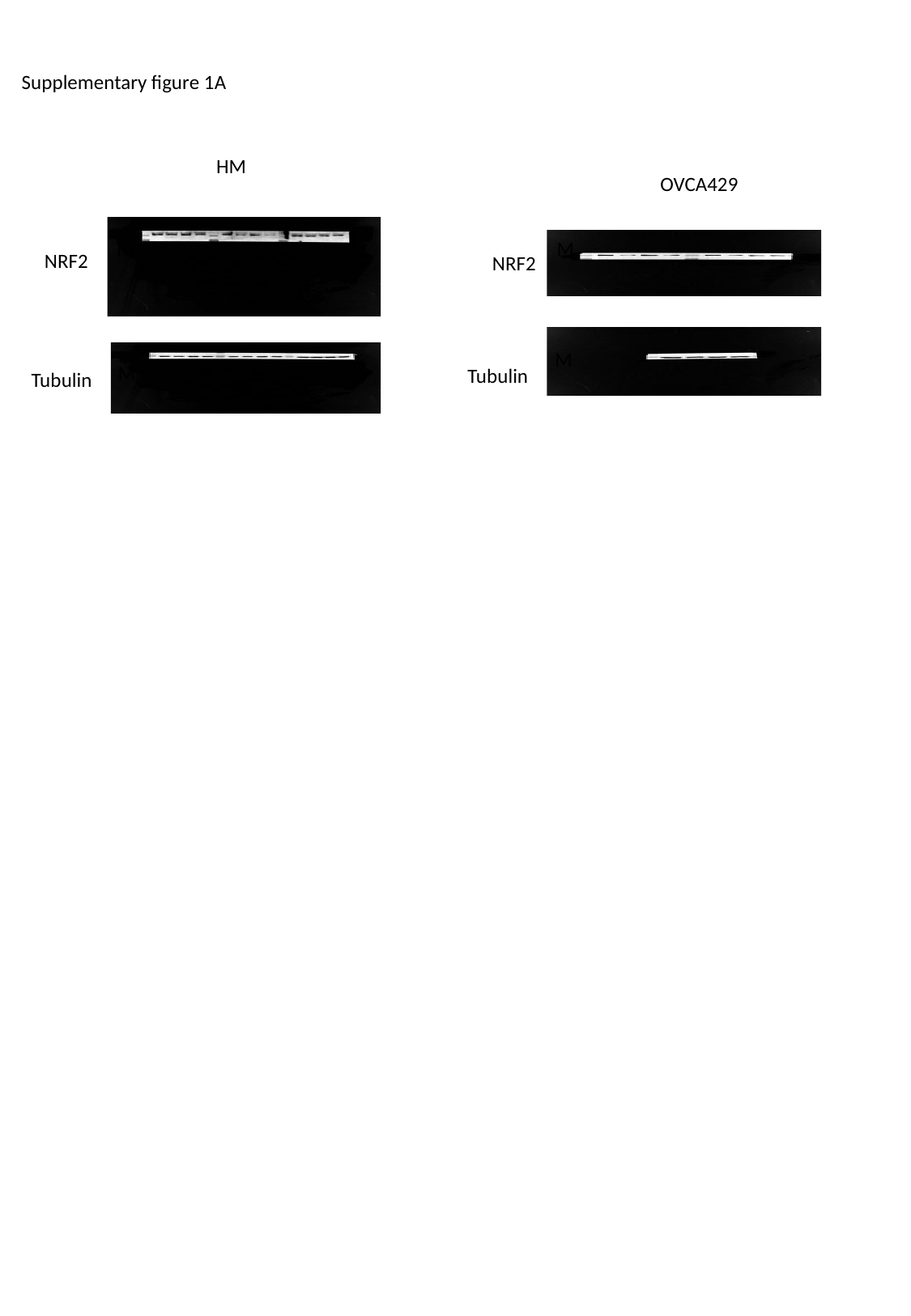

Supplementary figure 1A
HM
OVCA429
M
M
NRF2
NRF2
M
M
Tubulin
Tubulin
